# Impact of Smart Glasses on Patient Care Time in Emergency Medical Services Ambulance

**DOI:** 10.1017/S1049023X23006489

**Published:** 2023-12

**Authors:** Korakot Apiratwarakul, Lap Woon Cheung, Kamonwon Ienghong

**Affiliations:** 1. Department of Emergency Medicine, Faculty of Medicine, Khon Kaen University, Khon Kaen, Thailand; 2. Accident & Emergency Department, Princess Margaret Hospital, Kowloon, Hong Kong; 3. Department of Emergency Medicine, Li Ka Shing Faculty of Medicine, The University of Hong Kong, Pokfulam, Hong Kong

**Keywords:** ambulances, communication, health policy, response time, technology

## Abstract

**Introduction:**

The smart glasses were implemented as an innovative communication tool to enhance effectiveness in the field. The traditional mode of communication for Emergency Medical Services (EMS) was radio, which had significant restrictions, primarily that they were unable to transmit any visual data. To enhance efficiency, the smart glasses were used for a more accurate assessment of the condition of patients during transportation. At this time, however, no prior study has shown significant benefits of employing smart glasses into EMS.

**Study Objective:**

The primary objective of this study is to compare the duration of patient care in an ambulance between the use and non-use of smart glasses. The secondary objective is to identify the characteristics of data communication between the ambulance and the hospital.

**Methods:**

This retrospective study utilized data gathered from closed-circuit television (CCTV) in ambulances at Srinagarind Hospital, Thailand. The data were collected over a six-month period, specifically from July through December 2021. The study included two groups: the smart glasses group and no smart glasses groups, both used during EMS operations. The primary data collected focused on the duration of patient care in the ambulance. Additionally, the type and characteristics of data transfers via smart glasses during EMS operations were also recorded.

**Results:**

Out of the 256 EMS operations included in this study, 53.1% (N = 68) of the participants in the smart glasses group were male. The majority of operations were performed during the afternoon shift in both groups. The average patient care time in the smart glasses group was 10.07 minutes, while it was 5.10 minutes in the no smart glasses group (P <.001), indicating a significant difference. Visual data communication between the ambulance and the hospital via smart glasses predominantly involved vital signs (100.0%), physical examination (56.3%), and neurological examination (42.2%). The use of audio data from the hospital to the ambulance primarily included taking additional patient history (26.6%) and performing physical examinations (19.5%).

**Conclusion:**

The implementation of smart glasses in EMS operations resulted in an increase in patient care time in the ambulance. Furthermore, the use of smart glasses facilitated an effective channel of real-time two-way communication between the ambulance and the hospital.

## Introduction

Thailand’s Emergency Medical Services (EMS) are initiated with a call to the 1669 national emergency hotline from patients or other individuals; after that, the medical call center will dispatch either the nearest or most appropriate EMS units to provide emergency care.^1,2^ In the case of an emergency patient being put in an ambulance, the assessment and treatment process starts with taking into account patient history, measuring vital signs, giving physical examinations, and providing necessary treatment.^3^ Once the standard of emergency care has been implemented, the patient will be transported to the designated hospital via an emergency ambulance.

During the journey, the medical personnel on board the ambulance, such as doctors or nurses, are responsible for continuously assessing the patient’s condition. This involves observing the patient’s symptoms, conducting periodic questioning, and monitoring vital signs.^4^ The collected information is then communicated via radio to the destination hospital or the hospital command center. This communication serves to provide crucial information about incoming patients to the emergency department (ED). As a result, the ED can prepare the necessary personnel and medical equipment to promptly initiate treatment upon the patient’s arrival at the hospital.^5,6^

However, the process of transferring information from an ambulance to the destination hospital takes a lot of time. During this period, the EMS members who are responsible for reporting the information must take their eyes off the patient assessment due to the radio base station on Thai ambulances that are designed to be mounted on the front of the vehicle so that the reporter cannot continuously see and assess the patient’s condition. As a result, the diagnosis of changing symptoms of patients is sometimes delayed, resulting in worsening clinical conditions. Therefore, to shorten that period, smart glasses play an important role in emergency medical operations.^7,8^ The data can be visible from the ambulance to the hospital with real-time information. This means that EMS staff do not have to take their eyes off patients. Furthermore, smart glasses are used to get the orders directly from the hospital to the ambulance members in case of time-sensitive conditions of the patients, such as cerebrovascular disease, multiple traumas, and cardiac arrest. Doctors on duty at the destination hospitals can see the process of care on an ambulance and provide additional history directly to the team in the ambulance. The suggested treatment can also be provided to ambulance personnel in cases whose symptoms were not clear. The use of smart glasses in EMS is recognized as a form of online medical oversight, aimed at enhancing the efficiency and safety of services delivered to emergency patients. By enabling real-time communication and video streaming, smart glasses allow remote medical professionals to provide guidance, support, and oversight to EMS personnel on the scene.

The primary objective of this study was to compare the duration of patient care in the ambulance between smart glasses and no smart glasses used. The secondary outcome was to identify the type and characteristics of data transfers via smart glasses during EMS operations.

## Methods

### Design and Setting

This retrospective study was carried out utilizing various EMS units at Srinagarind Hospital, Thailand. The hospital is the primary center in the region for training emergency physicians and EMS technicians with 1,500 EMS (basic level) and 1,000 EMS (advanced level) operations carried out annually. In the EMS unit, there is a dispatch center which operates and controls six ambulances. The smart glasses were implemented in the EMS units starting in 2021. This equipment was new in the health care system. The EMS providers who used smart glasses were well-trained in the instruction of this equipment and no motion sickness was reported during the operations.

### Participants

All data on patients treated with an EMS ambulance during the study period and recorded in a closed-circuit television (CCTV) database were collected. Cases with incomplete data and cases where the patients were not found were excluded from this study. Furthermore, cases in which EMS members experienced motion sickness while wearing smart glasses were excluded from this study.

### Data Collection

Data were collected from July through December 2021. The data were extracted from four CCTVs, which were provided in the ambulances. In each EMS operation, the data were reviewed on a case-by-case basis in the process of EMS transport being carried out. The EMS practitioners proceeded to transfer the patient to a hospital via ambulance for specialized care and provided medical care during transportation. The primary data obtained were the duration of patient care in the ambulance, which was overseen by three emergency physicians, each with over ten-year experience in EMS. The type of data communication between ambulance and hospital via smart glasses was divided into visual and audio data.

### Definition

Transport time is defined as the period of time from when the patient has been placed in the ambulance and ends when the patient’s care is transferred to the destination hospital.

Patient care time is the sub-period of the transport time, defined as the duration during which EMS providers provide medical attention and care to a patient while they are only in the ambulance. It includes the time spent assessing the patient, administering treatments or interventions, monitoring vital signs, and addressing any immediate medical needs or emergencies.

### Sample Size

The sample size was calculated based on previous studies.^5^ Thus, the authors determined that a sample size of 256 would be required. The statistical analysis was performed with Khon Kaen University’s (Khon Kaen, Thailand) license for IBM SPSS for Windows version 27.0 (IBM Corp.; Armonk, New York USA). The categorical data obtained are presented as frequencies and percentages. Continuous data are presented using the means and standard deviations (SD). The chi-square test was used to assess the relationship between categorical variables. A two-tailed P <.05 was considered statistically significant.

### Smart Glasses

**Figure 1.**
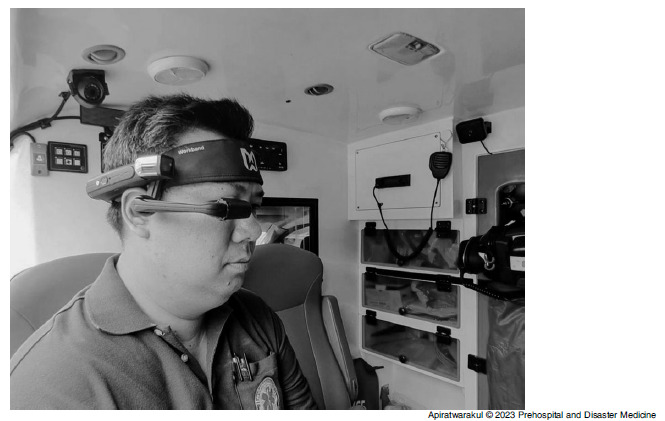
Smart Glasses on Ambulance.

The smart glasses were manufactured by The Real Wear Company (Vancouver, Washington USA). It was the HMT-1 model. Android 10.0 was the operating system, and it was connected via Wi-Fi 2.4 GHz and 5 GHz or Bluetooth Low Energy 4.1. The data were from smart glasses including visual and audio with real-time two-way communications. The EMS members who responded to those wearing smart glasses were the team leaders (doctor or nurse; Figure 1).

### Ethical Considerations

Ethical approval was provided by the Khon Kaen University Ethics Committee for Human Research (HE651049). Requirement for informed consent from the patients was waived since patient confidentiality protection had been guaranteed, as patients were not identified by name, but by a unique study number.

## Results

Over the period of the six-month study, 256 EMS operations were collected from the EMS CCTV database, the characteristics of which are shown in Table 1. A total of 53.1% (N = 68) were male in the smart glasses group. Operation times were most frequent in the afternoon shifts in both groups. In the smart glasses group, non-trauma patients accounted for 68.7% of all cases in the study.

**Table 1.** Characteristics of the Subjects (N = 256)

Feature	Category	**Smart Glasses** **N = 128**	**Non-Smart Glasses** **N = 128**	P Value
Gender, N (%)	Male	68 (53.1)	66 (51.6)	.864
Age (years)	Mean (SD)	52.1 (6.8)	51.7 (6.4)	.852
Operation Time, N (%)				.820
	Morning Shift	40 (31.3)	42 (32.8)	
	Afternoon Shift	54 (42.2)	55 (43.0)	
	Night Shift	34 (26.5)	31 (24.2)	
Telephone Triage Level, N (%)				.620
	Resuscitation	32 (25.0)	30 (23.4)	
	Urgency	96 (75.0)	98 (76.6)	
Type of Patients, N (%)				.632
	Trauma	40 (31.3)	38 (29.7)	
	Non-Trauma	88 (68.7)	90 (71.3)	

The average time from dispatch to vehicle en route (activation time) for the smart glasses and the no smart glasses groups were 1.02 minutes and 1.00 minutes, respectively (P = .442; Table 2). The average patient care time in the smart glasses group was 10.07 minutes, and the no smart glasses group was 5.10 minutes (P <.001).

**Table 2.** Comparing Service Times between Smart Glasses and Non-Smart Glasses Groups in the Ambulance

Time	**Smart Glasses (min)** **N = 128**		**Non-Smart Glasses (min)** **N = 128**		P Value
	Mean	95% CI	Mean	95% CI	
Activation Time	1.02	(0.80-1.07)	1.00	(0.92-1.07)	.442
Response Time	9.46	(9.01-10.20)	9.50	(9.06-10.30)	.520
Transport Time	12.01	(11.06-14.01)	12.20	(10.80-14.20)	.611
Patient Care Time	10.07	(9.10-10.70)	5.10	(3.47-6.20)	<.001^a^

a Statistical significance.

The type of visual data communication between the ambulance and hospital via smart glasses primarily accounted for vital signs (100.0%), physical examinations (56.3%), and neurological examinations (42.2%). In Table 3, the primary types of audio data conveyed from ambulance to hospital were gender, age, and chief complaint (100.0%). The study found that the type of audio data transmitted from the hospital to the ambulance showed a significant increase in obtaining patient history (26.6%) and conducting physical examinations (19.5%). This suggested that during the ambulance journey, medical personnel at the hospital provided important information related to the patient’s medical history and instructions regarding physical examinations to be performed.

**Table 3.** Characteristics of Data Communication via Smart Glasses (N = 128)

Feature	Category		Frequency	Percent
Visual	Vital Signs		128	100.0
	Physical Examination		72	56.3
	Neurological Examination		54	42.2
	Pupil Examination		12	9.4
Audio	From Ambulance to Hospital	Gender	128	100.0
		Age	128	100.0
		Chief Complaint	128	100.0
		Onset	116	90.6
		Time at Arrival Scene	98	76.6
		EMS Triage Level	96	75.0
		Provisional Diagnosis	120	93.8
	From Hospital to Ambulance	Patient’s History	34	26.6
		Physical Examination	25	19.5
		Immediate Treatment	20	15.6

Abbreviation: EMS, Emergency Medical Services.

## Discussion

This study focused on the implementation of smart glasses, a new technology that will increasingly be employed in Thailand’s EMS, in order to improve the efficiency of patient care while in the ambulance, and to assist in the capacity of doctors stationed at destination hospitals to transmit patient information and analyze symptoms.^9–11^ The Faculty of Medicine, Khon Kaen University has been using smart glasses on ambulances since 2021.

The traditional method of transferring information from ambulances to hospitals in Thailand without telemedicine systems is radio.^2,5,6^ This has been found to have many limitations, such as unclear radio communication, requiring a long time to make contact, confusion over codes used to communicate via radio communication, and no visual aids to help in the assessment of the patient’s physical examination. For these reasons, smart glasses technology has been adopted to solve such problems.^12^ The expectation is to more effectively convey information from ambulances, both audio and visual, to accurately assess symptoms and prepare for treatment at the ED.

According to the study, using smart glasses in ambulances increased patient care time more than not using them. The main reason was that the use of smart glasses required personnel stationed in the ambulance to monitor the patient at all times, as well as observe changes and transmit data of vital signs utilizing equipment installed on the ambulance. As a result, when there was a change in the patient’s condition, the personnel in the ambulance were able to assess the condition in a timely manner. Furthermore, the increased period of care for patients in the ambulances demonstrated that unnecessary time was effectively reduced because EMS members no longer had to rely solely on radio communication to report patient information, which was prone to errors. Therefore, the adoption of smart glasses technology in EMS will support more effective patient evaluation, diagnosis, and treatment with reduced duration of time. Moreover, the use of smart glasses did not affect the delay of the scene time and transport time due to the EMS members wear the equipment during the moving ambulance.

Types of communication data transmitted through smart glasses can be divided into two categories: visual and audio data. In the field of visual information, referral of information from the ambulance to the destination hospital with patient vital signs and physical examinations are provided to doctors stationed at the destination hospitals. In addition, if necessary, specific medical equipment at the ED will be prepared ahead of patient arrival.

In the field of audio data, there is a two-way communication route; from the ambulance to the hospital, and from the destination hospital is also transferred to the personnel in the ambulance. In accordance with the standards of EMS operations in Thailand, personnel on board the ambulance will report preliminary patient information consisting of gender, age, chief complaint, and onset, which is important information to the hospital. There is also a provisional diagnosis from EMS members in an ambulance.

The use of smart glasses in EMS creates an additional channel for the transfer of essential information from the destination hospital to the ambulance, especially for medical history, physical examinations, and treatment.^13,14^ In the case where initial diagnosis is not possible, further medical examination will be required, which has a positive effect. This allows the patient to be diagnosed quickly. In addition, in cases where resuscitation is needed, the doctor stationed at the destination hospital can also order immediate treatment in the ambulance. The procedure in the ambulance (chest compression, prehospital point-of-care ultrasound, and endotracheal intubation), with the guidance and monitoring from the staff at the hospital, will allow the EMS members to have more confidence to perform these procedures.^1,2,6^

Development of Thailand’s EMS by using modern technology has always and will continue to play an important role in public health policy. Efficient communication equipment and communication networks will change the treatment guidelines in ambulances to be more convenient, faster, and more efficient in response to the ever-growing needs of society, ensuring sustainable development, and increasing service safety records.^15,16^

## Limitations

This study analyzes operational data in the EMS through a CCTV database recorded through cameras installed on ambulances. It relies on experienced doctors who make judgment calls and analyze the duration time of EMS operations. In addition, efficient databases with clear audio and visual aids are an important factor in the data analysis.

The characteristics of EMS in each country vary in terms of personnel, vehicles, and treatment guidelines, so the use of information depends on such key factors.

## Conclusions

The implementation of smart glasses increased patient care time in EMS ambulances and created effective channels of real-time two-way communication between ambulance and hospital.
